# The future of fertility preservation for women treated with chemotherapy

**DOI:** 10.1530/RAF-22-0123

**Published:** 2023-05-08

**Authors:** Lauren R Alesi, Quynh-Nhu Nguyen, Jessica M Stringer, Amy L Winship, Karla J Hutt

**Affiliations:** 1 Department of Anatomy and Developmental Biology, Monash Biomedicine Discovery Institute, Monash University, Clayton, VIC, Australia; 2 Paediatric Integrated Cancer Service, VIC, Australia

**Keywords:** cancer, chemotherapy, fertility preservation, oncofertility, ovary, oocyte

## Abstract

Cytotoxic chemotherapies have been a mainstay of cancer treatment but are associated with numerous systemic adverse effects, including impacts on fertility and endocrine health. Irreversible ovarian damage and follicle depletion are the side effects of chemotherapy that can lead to infertility and premature menopause, both being major concerns of young cancer patients. Notably, many women will proceed with fertility preservation, but unfortunately existing strategies do not entirely solve the problem. Most significantly, oocyte and embryo freezing do not prevent cancer treatment-induced ovarian damage from occurring, which may result in the impairment of long-term hormone production. Unfortunately, loss of endogenous endocrine function is not fully restored by hormone replacement therapy. Additionally, while GnRH agonists are standard care for patients receiving alkylating chemotherapy to lessen the risk of premature menopause, their efficacy is incomplete. The lack of more broadly effective options stems, in part, from our poor understanding of how different treatments damage the ovary. Here, we summarise the impacts of two commonly utilised chemotherapies – cyclophosphamide and *cis*-diamminedichloroplatinum(II) (cisplatin) – on ovarian function and fertility and discuss the mechanisms underpinning this damage. Additionally, we critically analyse current research avenues in the development of novel fertility preservation strategies, with a focus on ferto-protective agents.

**Lay summary:**

Over the past few decades, advances in the detection and treatment of cancer have dramatically improved survival rates in young women. This means that ensuring patients have a high quality of life after cancer treatment has become a new priority. Therefore, it is important to understand and prevent any long-term negative side effects of cancer treatments, with infertility and early-onset menopause being major concerns for women receiving chemotherapy. The current fertility preservation options available to young women have significant limitations. Therefore, the identification of new approaches to protect fertility has been an intense topic of research in recent years. In this review, we provide information on the negative side effects of two commonly used chemotherapy drugs – cyclophosphamide and *cis*-diamminedichloroplatinum(II) (cisplatin) – on fertility, and discuss how they cause damage to the ovaries. We also critically analyse recent preclinical studies related to the development of new fertility preservation techniques.

## Introduction

Given that approximately 5% of women diagnosed with cancer worldwide are of reproductive age and cancer mortality rates are steadily falling (Bedoschi *et al.* 2016), addressing the off-target effects of cancer treatment on ovarian function, fertility and endocrine health has become a prominent issue. In fact, between 40% and 80% of female cancer survivors experience infertility post-treatment (Stern *et al.* 2006, Knapp *et al.* 2011). Moreover, many patients report this risk of post-treatment infertility to be equally as distressing as the initial cancer diagnosis (Logan *et al.* 2019). Consequently, understanding and mitigating these long-term, off-target consequences of cancer therapy on fertility must be prioritised.

Female fertility is governed by the quantity and quality of oocytes, which are stored in the ovary within primordial follicles. Cytotoxic chemotherapy, in addition to radiotherapy and surgery, has long been the mainstay of many cancer treatment regimens and is well documented to have detrimental off-target side effects on the ovaries, fertility and endocrine health (Bedoschi *et al.* 2016). Primordial follicle oocytes are exquisitely sensitive to genotoxic stress (i.e. DNA damage), and their depletion is accelerated in response to many exogenous insults, including chemotherapy treatment (Kerr *et al.* 2012*b*, Winship *et al.* 2018).

Exposure to certain chemotherapeutic drugs may induce temporary amenorrhea (cessation of menstruation) and subfertility in the short term or cause premature ovarian insufficiency (POI) in the long term (Codacci-Pisanelli *et al.* 2017). Indeed, exposure to gonadotoxic cancer treatment is the leading cause of POI in young girls and women (Byrne *et al.* 1992). POI is characterised by reduced ovarian endocrine function, complete amenorrhea and early menopause in women younger than 40 years of age, which results in irreversible infertility (Webber *et al.* 2016). This not only has the potential to significantly impact a patient’s mental health but is also associated with multi-system long-term physical and psychological sequelae due to the associated ovarian endocrine failure. These sequelae can significantly impact quality of life and may include impaired sexual health, increased risk of depression and anxiety and increased risk of cardiovascular disease and osteoporosis, among others (Faubion *et al.* 2015, Torrealday *et al.* 2017). Therefore, preventing ovarian damage associated with chemotherapy is not only important for protecting the fertility of female cancer survivors but also their overall long-term health and well-being.

Unfortunately, current fertility preservation options and techniques have some significant limitations. Embryo, oocyte and ovarian cortex cryopreservation are effective for many adult women but are expensive, invasive and not available to all patients. Moreover, these treatments do not actually mitigate ovarian damage and thus do not prevent POI. Notably, treatment with gonadotropin-releasing hormone (GnRH) agonists has been shown to reduce the risk of POI in women with breast cancer (Lambertini *et al.* 2018). However, these have not been definitively shown to protect the ovarian reserve or preserve fertility (Lambertini *et al.* 2019). Therefore, the investigation of novel fertility preservation strategies is a critical area of research.

## Off-target impacts of chemotherapy on ovarian function

Numerous classes of chemotherapy exist, including alkylating agents, platinum-based (alkylating-like) agents, anti-tumour antibiotics, anti-metabolites, vinca alkaloids, topoisomerase inhibitors and other miscellaneous agents. These different classes of chemotherapeutic agents have diverse mechanisms of action and, hence, varying degrees of ovarian toxicity (ovotoxicity), which have been reviewed extensively (Bedoschi *et al.* 2016, Spears *et al.* 2019, Alesi *et al.* 2021). Briefly, the most ovotoxic class is the alkylating agents, such as cyclophosphamide, followed by platinum-based compounds, such as *cis*-diamminedichloroplatinum(II) (cisplatin), and, lastly, the anthracycline antibiotic doxorubicin. However, other agents, such as the anti-metabolite 5-fluorouracil, have been demonstrated to have moderate, short-term ovotoxicity (Lambouras *et al.* 2018, Stringer *et al.* 2018). Additionally, it is important to consider that the ovotoxicity of chemotherapies is dependent on dose and frequency of treatment and is likely exacerbated in multi-dose and combination regimens.

Characterising the ovotoxicity of chemotherapies remains an active area of research, with several recent studies of previously overlooked agents revealing detrimental impacts on ovarian function (reviewed recently byAlesi *et al.* 2021, updates in Table 1). This new body of research highlights the requirement for more rigorous investigation of the ovotoxicity of both existing and new cancer therapies to adequately inform clinicians and patients of the risks to fertility posed by treatment. However, as cyclophosphamide and cisplatin are the most widely studied agents with respect to ovarian function, they will be the major focus of this review hereafter.

**Table 1:** Update on ovotoxicity of chemotherapies.

Agent	Class	Mechanism of action	Evidence of ovotoxicity	Degree of ovotoxicity	Reference
Paclitaxel	Taxane	Induces microtubule destabilisation	Single-dose (30 mg/kg) paclitaxel decreases the number of healthy antral follicles *in vivo* in mice, without impacting primordial follicles	Mild, short term	Ma *et al.* (2020)
5-FU	Anti-metabolite	Interferes with DNA replication		Varies from mild, short term to high, long term, depending on the dose and frequency	
			Multi-dose 5-FU (50 mg/kg/day for 4 days) does not alter primordial follicle number *in vivo* in mice but depletes growing follicles. Additionally, oocyte maturation and early embryo development is impaired *in vitro*		Naren *et al.* (2021)
			Single-dose (450 mg/kg) 5-FU significantly reduces the number of follicles in all classes *in vivo* in mice, including primordial follicles		Almeida *et al.* (2021)
Actinomycin D	Antibiotic	Interferes with DNA transcription	Actinomycin D disrupts spindle assembly and chromatin condensation in mouse and human oocytes *in vitro.* Additionally, oocyte maturation is impaired in mouse oocytes *in vitro*	Mild, short term	Li *et al.* (2021)
Irinotecan (CPT-11)	Topoisomerase inhibitor	Interferes with DNA replication	Single-dose (100 mg/kg) irinotecan significantly decreases primordial follicles and serum AMH *in vivo* in mice	Moderate, long term	Levi *et al.* (2022)

CPT-11, 7-ethyl-10-[4-(1-piperidino)-1-piperidino] carbonyloxycamptothecin; 5-FU, 5-fluorouracil.

Cyclophosphamide is a nitrogen mustard derivative used to treat a variety of cancers that commonly affect paediatric and reproductive-age female patients, including Hodgkin’s and non-Hodgkin’s lymphoma, breast cancer, ovarian cancer and small cell lung cancer, among others (Ogino & Prasanna 2020). Once metabolised in the liver, cyclophosphamide is converted into its active alkylating metabolites, which include phosphoramide mustard and acrolein. These form crosslinks both within and between DNA strands, resulting in double-stranded DNA breaks, which, if irreparable, culminate in apoptosis of the target cell (Chu & Rubin 2018).

*C*isplatin is a platinum-based chemotherapy that is widely used and effective in the treatment of numerous cancer types. These include ovarian cancer, breast cancer, cervical cancer, lung cancer (both small-cell and non-small cell), brain cancer and neuroblastoma, all of which affect paediatric and reproductive-age females (Dasari & Tchounwou 2014, Franasiak & Scott 2016). Sometimes described as an alkylating-like agent, cisplatin is able to crosslink DNA and form both inter- and intra-strand adducts (Dasari & Tchounwou 2014). This causes DNA damage, blocks cell division, prevents induction of DNA repair and, usually, leads to apoptosis of the target cell. Another critical component of cisplatin-induced cytotoxicity is the induction of oxidative stress *via* the production of reactive oxygen species, predominantly within mitochondria, leading to mitochondrial dysfunction and induction of apoptosis by either the intrinsic or the extrinsic pathway, independent of DNA damage (Marullo *et al.* 2013, Dasari & Tchounwou 2014).

Since cyclophosphamide metabolites and cisplatin are capable of directly interacting with DNA, thereby leading to the induction of double-stranded breaks and inhibition of DNA replication, they are both considered to act in a cell cycle non-specific manner (Sun *et al.* 2021). However, susceptibility to damage can vary across the cell cycle, with sensitivity to cisplatin peaking at G_1_ just prior to the onset of DNA replication (Shah & Schwartz 2001). Therefore, even meiotically arrested cells, such as the oocytes within primordial follicles, are susceptible to cyclophosphamide- or cisplatin-induced damage.

## Evidence of cyclophosphamide- and cisplatin-induced ovarian damage

The deleterious effects of cyclophosphamide and cisplatin exposure on ovarian function are well established. Despite this, studies investigating the effect of single-agent cyclophosphamide in human ovarian tissue are surprisingly limited. Similarly, given that cisplatin is rarely administered as a single agent, there is also very limited data available on the precise impact of cisplatin alone on ovarian function in humans. Thus, the vast majority of knowledge regarding the ovotoxicity of these chemotherapies has been obtained from animal studies, particularly those conducted in rodents.

The consequences of cyclophosphamide and cisplatin exposure on ovarian function fall into three major categories: (i) impacts on primordial follicles and, thus, long-term fertility; (ii) impacts on growing follicles and, thus, short-term fertility; and (iii) impacts on ovarian stroma and vasculature. Additionally, recent evidence suggests that these agents may also impact upon oocyte mitochondrial function, though the consequences of this are yet to be fully elucidated (Malott & Luderer 2021, Wang & Hutt 2021, Zhang *et al.* 2021).

### Impacts on primordial follicles and long-term fertility

Exposure to cyclophosphamide or cisplatin is highly detrimental to primordial follicles and can permanently impact fertility in the long term. In fact, numerous animal studies conducted over the past several decades have documented a significant depletion of the primordial follicle pool in response to cyclophosphamide treatment (Mattison *et al.* 1981, Shiromizu *et al.* 1984, Kalich-Philosoph *et al.* 2013, Nguyen *et al.* 2018, Bellusci *et al.* 2019, Nguyen *et al.* 2019, Salian *et al.* 2020). Similarly, many *in vitro* and *in vivo* studies in mice published in recent years have demonstrated that cisplatin is also highly toxic to primordial follicles (Gonfloni *et al.* 2009, Chang *et al.* 2015, Yuksel *et al.* 2015, Rossi *et al.* 2017, Nguyen *et al.* 2018, Nguyen *et al.* 2019).

In adult mice, a single, high dose of cyclophosphamide (300 mg/kg) or cisplatin (5 mg/kg) is sufficient to eliminate the vast majority of the primordial follicle population within 24 h *in vivo* (Nguyen *et al.* 2019). Of those remaining, half are morphologically abnormal, which is indicative of imminent apoptosis (Nguyen *et al.* 2019). By 5 days post-treatment, approximately 5% and 25% of the primordial follicle pool remain following cyclophosphamide (300 mg/kg) and cisplatin (5 mg/kg) treatment, respectively (Nguyen *et al.* 2018). Additionally, lower multi-dose cyclophosphamide (100 mg/kg × 6) is also detrimental to ovarian function in mice *in vivo*, with very few primordial follicles remaining 4 weeks post-final treatment (Kim & You 2021). These studies clearly demonstrate the ovotoxicity of these alkylating agents.

As a consequence of the significant depletion to the primordial follicle pool, there is a dramatic reduction in the number of mature oocytes collected after superovulation, pregnancy rate, age at last litter, average number of pups per litter and total number of litters per female in cyclophosphamide or cisplatin-treated mice (Kerr *et al.* 2012*b*, Zhang *et al.* 2015, Nguyen *et al.* 2018, Roness *et al.* 2019, Sonigo *et al.* 2019, Huang *et al.* 2020, Salian *et al.* 2020, Kim & You 2021). These data indicate a significant reduction of fertility and the overall fertile life span in response to cyclophosphamide or cisplatin treatment. Interestingly, in a recent study, no gross abnormalities were reported in any offspring born from either cyclophosphamide- or cisplatin-treated mice (Nguyen *et al.* 2018). This data may suggest that the quality of the few remaining primordial follicles not eliminated by apoptosis is preserved or perhaps that any DNA damage was able to be repaired within the oocytes of those follicles. Supporting this hypothesis, it was recently demonstrated that primordial follicles can effectively repair DNA damage following cisplatin exposure *via* homologous recombination in apoptosis-resistant (*Puma^–/–^*) mice (Nguyen *et al.* 2021).

Although limited, there is also direct evidence of cyclophosphamide- and cisplatin-induced toxicity to primordial follicles in humans. A study using human fetal ovarian xenografts in mice demonstrated that cyclophosphamide treatment significantly depleted the primordial follicle population (Meng *et al.* 2014). Another study utilising cultured human ovarian tissue treated with metabolites of cyclophosphamide found similar results (Lande *et al.* 2017). *In vitro* studies utilising cultured human ovarian cortical pieces and cultured human granulosa cells have also revealed significant decreases in primordial follicles and steroid hormone production and a significant increase in granulosa cell apoptosis in response to cisplatin (Bildik *et al.* 2015, Yuksel *et al.* 2015, Bildik *et al.* 2018). Together, these data suggest that the negative impacts on fertility in female cancer survivors, who received cyclophosphamide and/or cisplatin, are likely to be due to a depletion in primordial follicles.

### Impacts on growing follicles and short-term fertility

Cyclophosphamide or cisplatin exposure can also impair growing follicle health and survival. Although transient, these effects can still interfere with short-term fertility, pregnancy success and offspring health. Toxicity to growing follicles has been reported in humans, with diminished antral follicle count and a dramatic reduction of circulating hormones produced by growing follicles (anti-Müllerian hormone (AMH) and inhibin B) following treatment with either cyclophosphamide-containing regimens or other alkylators (Anderson *et al.* 2006, van den Berg *et al.* 2018). However, in rodent studies, evidence of toxicity to growing follicles is conflicting.

Following cyclophosphamide or cisplatin exposure, significant depletion of the growing follicle pool – primarily in the early (primary and secondary) stages – has been reported in rats (Jarrell *et al.* 1991, Yuksel *et al.* 2015), although reports in mice following cyclophosphamide or cisplatin exposure are varied. Some studies describe significant depletion of only primary and/or secondary follicles (Kalich-Philosoph *et al.* 2013, Nguyen *et al.* 2018, Nguyen *et al.* 2019, Bellusci *et al.* 2019), whereas other studies are unable to detect any depletion altogether (Mattison *et al.* 1981, Rossi *et al.* 2017). Some other papers report an increase in the early growing follicle pool following cyclophosphamide or cisplatin exposure (Kalich-Philosoph *et al.* 2013, Chang *et al.* 2015, Sonigo *et al.* 2019). The discrepancy in reports may be explained by differences in follicle quantification methods or by differences in the interpretation of follicle quantification. Two of the three studies reporting a significant increase in the growing follicle pool found an increased ratio of primordial to growing follicles (Chang *et al.* 2015, Sonigo *et al.* 2019) rather than looking at the follicle numbers. An increased ratio of growing follicles may be caused by the depletion of primordial follicles by cyclophosphamide or cisplatin treatment rather than an increase in the number of growing follicles. Nevertheless, there appears to be a difference in sensitivity to cyclophosphamide or cisplatin exposure between primordial and growing follicles. This may be due to growing follicles having a higher threshold for apoptosis induction compared to primordial follicles or may reflect, perhaps, a greater capacity for DNA repair.

Irrespective of whether the quantity of growing follicles is decreased, increased or unaffected, exposure to cyclophosphamide or cisplatin in the short term does appear to impact the quality of growing and mature oocytes. Early studies indicated evidence of non-disjunction and increased chromosomal aberrations in mature, metaphase-II (MII) oocytes collected from cyclophosphamide-treated mice (Hansmann 1974, Hansmann & Probeck 1979). More recently, disrupted microtubule assembly, spindle structure and chromosome alignment have been reported in mouse MII oocytes exposed to cyclophosphamide both *in vivo* or *in vitro* (Jeelani *et al.* 2017, Del Castillo *et al.* 2021). Another recent study found similar results in mouse MII oocytes exposed to carboplatin (a cisplatin analogue) *in vitro* (Zhou *et al.* 2019). Moreover, poor oocyte quality is also reflected by elevated rates of fetal malformations and resorptions in pregnancies established 1 week post-cyclophosphamide treatment (Meirow *et al.* 2001). This suggests that growing follicles, although appearing to have a higher threshold for apoptosis induction, may not be able to efficiently repair the DNA damage or that organelles and/or maternal factors (proteins and RNA laid down in the oocytes during folliculogenesis) were compromised. This decrease in oocyte quality could result in detrimental effects on subsequent embryos formed, negatively impacting embryo implantation, survival and offspring health in pregnancies established shortly after chemotherapy.

### Impact on ovarian stroma and vasculature

The ovarian stroma and vasculature have an important role in coordinating ovarian steroid production (particularly oestradiol and testosterone) and supporting both the health of the ovarian reserve and normal follicular development (Bedoschi *et al.* 2016). In addition to a direct impact on ovarian follicles, cyclophosphamide and cisplatin exposure can cause stromal and vascular damage (Nicosia *et al.* 1985, Marcello *et al.* 1990, Meirow *et al.* 2007, Oktem & Oktay 2007*b*, Saleh *et al.* 2015, Luo *et al.* 2017, Pascuali *et al.* 2018). Moreover, a recent study examined the impact of phosphoramide mustard (a cyclophosphamide metabolite) and cisplatin on various ovarian somatic cell types in cultured prepubertal mouse ovaries, including pre-granulosa cells, pre-thecal cells and ovarian surface epithelium (Marcozzi *et al.* 2019). Both phosphoramide mustard and cisplatin exposure had similar effects, inducing extensive DNA damage and cell cycle arrest in these cell types (Marcozzi *et al.* 2019). Altogether, these effects may impact ovarian endocrine function whilst also indirectly impacting follicular development.

## Mechanisms of chemotherapy-induced ovarian damage

In order to identify new targets for fertility preservation, it is important to understand the mechanisms by which chemotherapies induce ovarian damage. However, the precise molecular mechanisms by which cyclophosphamide and cisplatin exert ovarian damage and deplete the ovarian reserve are not fully understood. Several putative mechanisms have been proposed to cause primordial follicle loss, and the relative contributions of these to chemotherapy-induced ovarian reserve depletion are still under active debate. Overall, proposed mechanisms fall into two major categories: (i) direct oocyte damage and apoptosis of primordial follicles and (ii) accelerated primordial follicle activation and ‘burnout’. A third factor may be induction of oxidative stress *via* the production of reactive oxygen species, which is thought to mediate stromal and vascular damage (Szymanska *et al.* 2020).

### Direct DNA damage and apoptosis

There is a large body of evidence to suggest that direct apoptosis of primordial follicles is likely the primary mechanism of cyclophosphamide- and cisplatin-induced primordial follicle loss (Plowchalk & Mattison 1992, Oktem & Oktay 2007*a*, Gonfloni *et al.* 2009, Petrillo *et al.* 2011, Kerr *et al.* 2012*a*, Kim *et al.* 2013, Morgan *et al.* 2013, Bellusci *et al.* 2019, Luan *et al.* 2019, Nguyen *et al.* 2019). For example, several recent *in vivo* mouse studies have demonstrated that primordial follicles sustain widespread DNA damage following cyclophosphamide or cisplatin exposure in the form of double-stranded breaks, predominantly within the oocyte (Bellusci *et al.* 2019, Nguyen *et al.* 2019, Yang *et al.* 2021). This occurs as early as 8 h after a single, high dose of cyclophosphamide or cisplatin (Nguyen *et al.* 2019) but may persist up to 24 h after low-dose cyclophosphamide treatment (Yang *et al.* 2021). This is evidenced by the presence of phosphorylated H2A histone family member X (γH2AX) staining in approximately half of primordial follicle oocytes (Nguyen *et al.* 2019). Moreover, terminal deoxynucleotidyl transferase dUTP nick-end labelling (TUNEL)-positive primordial follicle oocytes were detected at both 8- and 24-h post-treatment, which is indicative of apoptosis and imminent follicle atresia (Nguyen *et al.* 2019).

**Figure 1:**
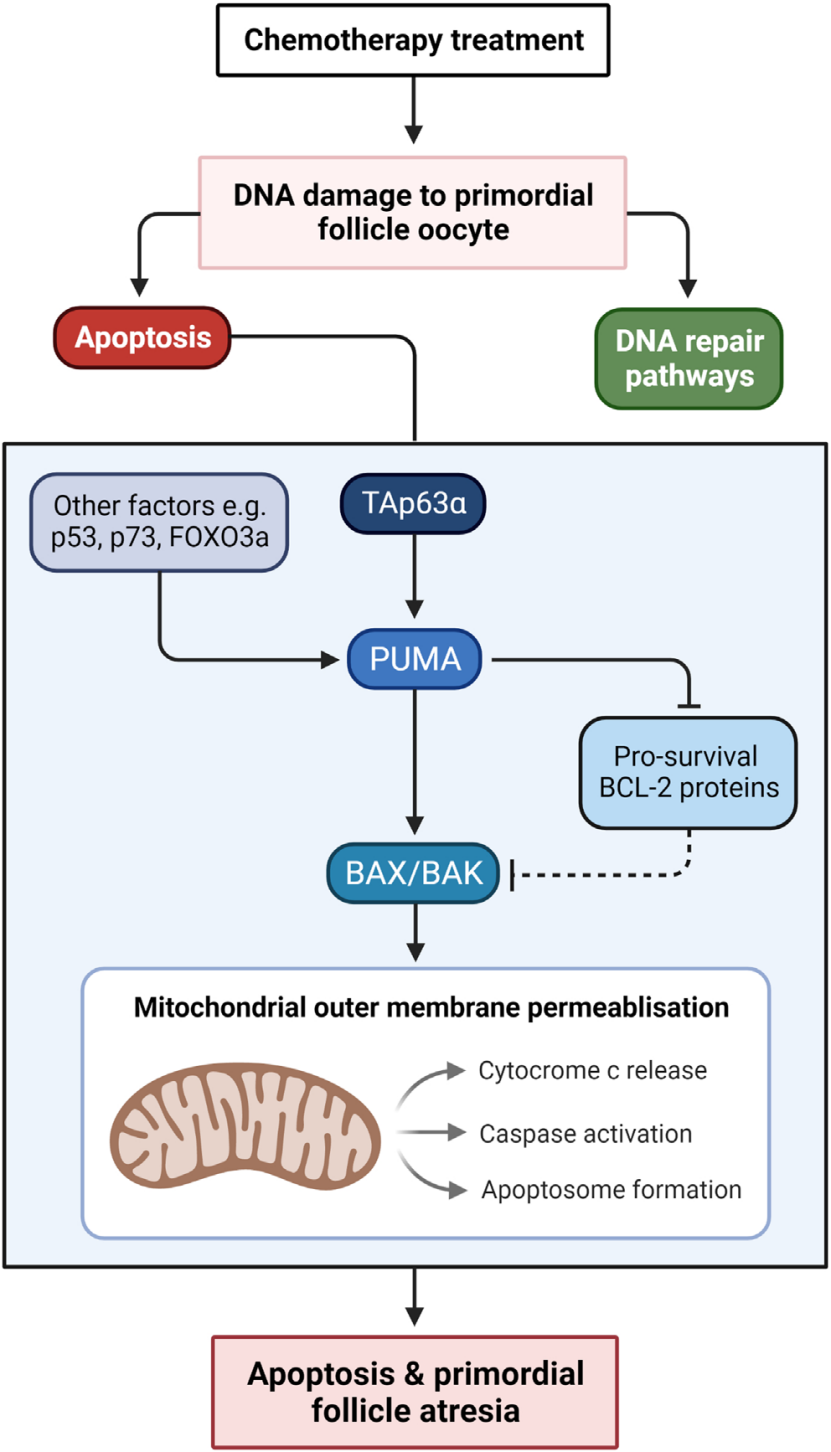
Direct DNA damage and apoptosis of primordial follicles following chemotherapy treatment. PUMA is the pro-apoptotic protein that mediates primordial follicle apoptosis in response to chemotherapy. In response to chemotherapy-induced DNA damage, PUMA is activated either by TAp63 or by other factors, such as p53, p73 or FOXO3a. Then, PUMA unleashes BAX and BAK either directly or indirectly by inhibiting the pro-survival BCL-2 proteins – which normally inhibit BAX and BAK. Subsequently, mitochondrial outer membrane permeabilisation occurs – the apoptotic ‘point of no return’ – causing cytochrome c release, caspase activation and formation of the apoptosome. Ultimately, this results in the apoptosis of the oocyte and atresia of the primordial follicle.

Shortly after the formation of double-stranded DNA breaks, ataxia–telangiectasia-mutated (ATM) kinase is activated. ATM can initiate DNA repair pathways but more commonly will phosphorylate and activate TAp63α (Suh *et al.* 2006, Livera *et al.* 2008) (Fig. 1). Subsequently, the pro-apoptotic BH3-only family members – which include PUMA and NOXA – are transcriptionally activated (Kerr *et al.* 2012*b*). Interestingly, activation of PUMA can also be mediated by other factors such as p53, p73 (another p53 homologue) and forkhead box O3a (FOXO3a), independent of transactivated p63 α (TAp63α; a p63 isoform and p53 homologue (Yu & Zhang 2008). PUMA (and to a lesser extent, NOXA) can induce apoptosis by binding and inactivating the pro-survival B-cell lymphoma 2 (BCL-2) proteins, resulting in the activation of BCL-2-associated X protein (BAX) and BCL-2 homologous antagonist killer (BAK) (Aubrey *et al.* 2018). PUMA is the more potent inducer of apoptosis, as it binds to all five of the pro-survival BCL-2 proteins with high affinity and can also directly interact with BAX (Youle & Strasser 2008). Upon activation of BAX and BAK, mitochondrial outer membrane permeabilisation occurs, which is regarded as the ‘point of no return’ in apoptosis. This results in cytochrome c release, caspase activation, and formation of the apoptosome, causing apoptosis of the oocyte and subsequent atresia (death) of the primordial follicle (Aubrey *et al.* 2018).

Prevention of apoptosis, through either genetic knockout of apoptotic genes or small-molecule inhibitors of apoptotic proteins, prevents primordial follicle depletion in response to chemotherapy (Nguyen *et al.* 2018, Luan *et al.* 2019). In this regard, a recent study utilising apoptosis-resistant (*Puma^–/–^*) mice demonstrated that when rescued from apoptosis, primordial follicles exposed to cisplatin are able to efficiently repair DNA damage *via* the homologous recombination pathway (Nguyen *et al.* 2021). This was evidenced by positive RAD51 staining in oocytes of primordial follicles 8 h post-treatment and complete resolution of γH2AX foci by 5 days. Together, these data strongly support direct damage as a key mechanism of oocyte depletion due to cyclophosphamide and cisplatin. Thus, preventing apoptosis of primordial follicles in response to chemotherapy represents a promising avenue for fertility preservation research.

### Accelerated primordial follicle activation and ‘burnout’

**Figure 2:**
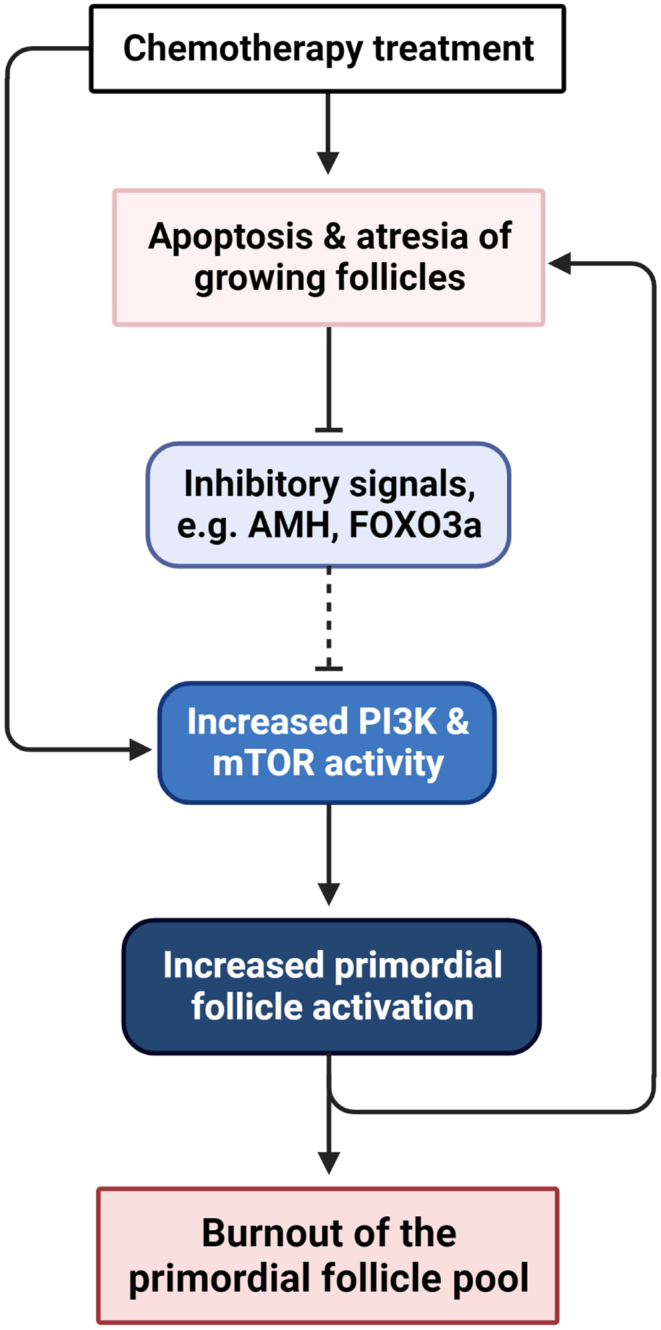
Overactivation and ‘burnout’ of primordial follicles following chemotherapy treatment. The ‘burnout’ theory states that chemotherapy treatment causes premature depletion of the primordial follicle pool *via* accelerated activation of primordial follicles. Chemotherapy treatment causes DNA damage in growing follicles, resulting in apoptosis and atresia of the growing follicle pool. Subsequently, this results in accelerated activation of the primordial follicle pool through increased activation of phosphoinositide 3-kinase (PI3K) and mechanistic target of rapamycin (mTOR) signalling. This may occur directly, or indirectly, *via* loss of inhibitory signals such as AMH (anti-Müllerian hormone) or FOXO3a (forkhead box O3a). These newly activated growing follicles are then susceptible to chemotherapy-induced damage, which leads to a cycle of accelerated activation that ultimately causes a burnout of the primordial follicle pool.

It is also recently been proposed that primordial follicle depletion might occur due to accelerated primordial follicle activation. Termed ‘burnout’, this theory states that cyclophosphamide and cisplatin only induce DNA damage and apoptosis of growing follicles not primordial follicles (Kalich-Philosoph *et al.* 2013, Chang *et al.* 2015, Roness *et al.* 2019). By rapidly depleting growing follicles, it is proposed that factors which normally regulate or suppress primordial follicle activation are impaired. These include phosphatase and tensin homolog, FOXO3a and AMH, among others (Dunlop & Anderson 2014). This results in a subsequent upregulation of factors which promote primordial follicle activation, such as the phosphoinositide 3-kinase (PI3K) and mechanistic target of rapamycin (mTOR) pathways plus their downstream regulators (Chen *et al.* 2020). Once these follicles are recruited from the resting pool, an increase of early growing (primary and secondary) follicles is then expected. Ultimately, since primordial follicle activation is not reversible, this increased activation is thought to result in an exhaustion, or ‘burnout’, of the remaining primordial follicle population (Fig. 2). Since chemotherapy regimens often consist of multiple doses, this could continually deplete the growing follicle population and indirectly the primordial follicle pool.

However, recent literature on whether cyclophosphamide and cisplatin induce overactivation of primordial follicles is conflicting. A study utilising cultured human cortical samples treated *in vitro* with cyclophosphamide metabolites demonstrated significantly decreased proportions of primordial follicle and increased proportions of growing follicles, with no evidence of apoptosis found (Lande *et al.* 2017). It is unclear whether the changes in follicle proportions were due to increased death of primordial follicles or increased activation. However, a study examining ovaries from women treated with alkylating agents 1–6 months prior to collection reported a decrease in in primordial follicle numbers and an increase in the absolute number of growing follicles (Shai *et al.* 2021). Moreover, FOXO3a immunostaining indicated a possible decrease in the percentage of FOXO3a-positive primordial follicle oocytes, which could indicate increased follicle activation (Shai *et al.* 2021). On the other hand, continuous treatment with either 2 mg/kg or 5 mg/kg cisplatin was shown to deplete primordial follicles *in vivo* in mice, without evidence of accelerated primordial follicle activation (Eldani *et al.* 2020). Additionally, using a murine human ovarian xenograft model, single-cell RNA sequencing of laser-captured primordial follicle oocytes found no difference in the expression of *Akt*, *rpS6* or *Foxo3a* 12 h following cyclophosphamide treatment (Titus *et al.* 2021). DNA damage and apoptosis were also detected in primordial follicles, as demonstrated by significant increases in γH2AX and active caspase-3 staining.

Overall, the current body of literature suggests that the precise contribution of accelerated primordial follicle activation to chemotherapy-induced follicle depletion remains unclear and is yet to be firmly established. Nevertheless, it is likely that this is contributing to primordial follicle loss in response to chemotherapy to some degree albeit potentially to a lesser extent than direct apoptosis.

## Existing fertility preservation options

For paediatric, adolescent or reproductive-age females undergoing cancer treatment, fertility preservation methods are available but are associated with some important limitations (Table 2). These existing options fall into two categories: (i) cryopreservation (i.e. freezing), which includes cryopreservation of oocytes, embryos or ovarian tissue and (ii) suppression of ovarian function using GnRH agonists (Rodriguez-Wallberg & Oktay 2014).

**Table 2:** Summary of existing fertility preservation options and their respective advantages and limitations.

Category/technique	Advantages	Limitations
Cryopreservation		
Oocyte cryopreservation		
	Established technique in post-pubertal females	Invasive and requires ovarian hormone stimulation
	Does not require sperm and thus is more accessible to unpartnered females	Can delay the commencement of cancer treatment
		Unavailable to prepubertal girls
		Requires subsequent IVF
		Does not prevent ovarian damage from occurring and thus will not prevent POI
Embryo cryopreservation		
	Established technique in post-pubertal females	Invasive and requires ovarian hormone stimulation
		Can delay the commencement of cancer treatment
		Unavailable to prepubertal girls
		Requires sperm, from either a partner or a donor
		Does not prevent ovarian damage from occurring and thus will not prevent POI
Ovarian tissue cryopreservation		
	Available to prepubertal girls	Invasive
	Does not require ovarian hormone stimulation	Limited success rates
	Does not significantly delay the commencement of cancer treatment	Remains experimental in prepubertal patients
	No longer considered experimental in adult patients	Risk of cancer reintroduction upon transplant
		Unable to fully compensate for endogenous ovarian function and thus will not likely prevent POI
Suppression of ovarian endocrine function	Does not delay the commencement of cancer treatment	Supporting evidence is conflicting and limited
		Does not prevent ovarian damage from occurring and thus will not prevent POI
GnRH agonists		
Oral contraceptives		

GNRH, gonadotropin-releasing hormone; IVF, *in vitro* fertilisation; POI, premature ovarian insufficiency.

### Oocyte and embryo cryopreservation

Oocyte and embryo cryopreservation have long been the backbone of female fertility preservation in adult cancer patients. These techniques both initially involve the retrieval of mature MII oocytes prior to commencement of chemotherapy treatment. These oocytes are either frozen after collection or undergo *in vitro* fertilisation (IVF) with partner or donor sperm to form embryos, which are then frozen. However, this invasive procedure first requires hormone stimulation to produce multiple mature oocytes, which in some cases will require a delay in the commencement of treatment that may not be feasible. Moreover, oocyte retrieval and IVF are inherently expensive and thus are not accessible to all patients (Quinn *et al.* 2008). Critically, these options do not prevent ovarian damage from occurring and thus do not prevent POI or its clinical sequelae.

### Ovarian tissue cryopreservation

Ovarian tissue cryopreservation (OTCP) involves the collection of small slices of ovarian cortex usually *via* laparoscopic surgery prior to the commencement of cancer treatment. This tissue is then cryopreserved and retransplanted (i.e. autografted) at a later time (Quinn *et al.* 2008). An increasing number of livebirths have been reported following reimplantation of cryopreserved ovarian tissue, and, as such, this technique is no longer considered experimental in adult women in many countries (Donnez & Dolmans 2015). However, this technique is invasive, and, to date, success rates have been modest (Forman 2018, Andersen *et al.* 2019). Moreover, the transplantation of the ovarian tissue back into the patient carries a risk of reintroducing malignant cells from the primary tumour, especially for haematologic malignancies (Dolmans *et al.* 2010, Bittinger *et al.* 2011, Gook & Edgar 2019).

For prepubertal girls, fertility preservation remains a challenge. Cryopreservation of oocytes or embryos is not possible; thus, cryopreservation of ovarian tissue is the only available option. As well as the issues with OTCP already described above, to date no livebirths have been reported following the reimplantation of prepubertally harvested ovarian tissue into adult survivors of childhood cancer (Quinn *et al.* 2008, Forman 2018, Kim *et al.* 2018). Therefore, a non-invasive pharmacological ovarian protectant (i.e. ferto-protective agent) would provide extraordinary benefit to female cancer patients from childhood through reproductive age and would clearly be preferable to additional, urgent, surgical intervention around the time of cancer diagnosis.

### GnRH agonists

**Figure 3:**
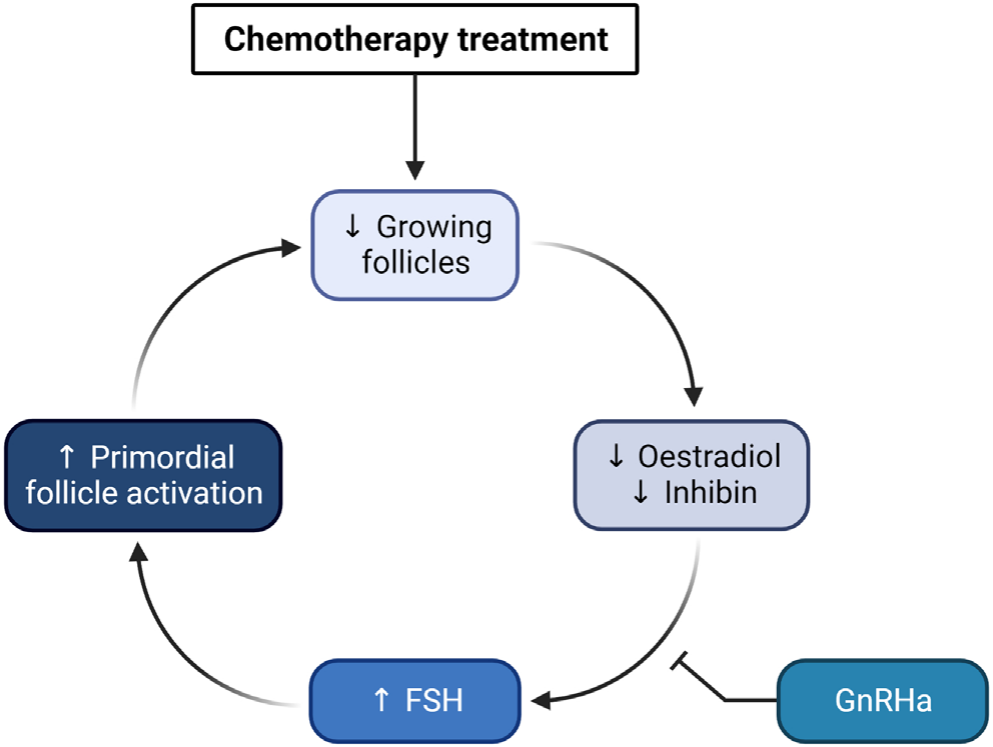
Rationale behind the use of GnRH agonists for fertility preservation. The rationale behind the use of GnRH agonists is that chemotherapy treatment results in a depletion of growing follicles within the ovary. Loss of these hormone-producing follicles causes a decrease in oestradiol and inhibin levels, which in turn results in an increase in follicle-stimulating hormone (FSH) levels *via* negative feedback. This increase in FSH is thought to accelerate recruitment of primordial follicles from the resting pool. Administration of GnRH agonists (GnRHa) prevents this increase in FSH levels which, by extension, is thought to prevent this overactivation of primordial follicles.

GnRH agonists are currently the only non-invasive clinical agents aimed at preserving fertility, although their efficacy in preventing chemotherapy-induced POI has been widely debated (Partridge 2012, Loren *et al.* 2013, Moore *et al.* 2015). The use of these agents is based on the idea that by suppressing ovarian endocrine function, the ovary may be protected from the ovotoxic effects of chemotherapy (Fig. 3). This hypothesis was developed following clinical observations that post-pubertal females are more vulnerable to chemotherapy-induced POI than prepubertal girls (Meirow & Nugent 2001). However, this theory is problematic from a mechanistic standpoint, since primordial follicles do not express gonadotrophin receptors, and DNA damage can still occur to oocytes stored within primordial follicles regardless of pituitary hormone status (Oktay *et al.* 1997, Méduri *et al.* 2002). Indeed, the literature on the clinical efficacy of GnRH agonists for ovarian protection is conflicting, and many prospective randomised trials have failed to clearly demonstrate a beneficial effect for preserving fertility (Partridge 2012). However, a recent meta-analysis reported improved spontaneous pregnancy rates in breast cancer patients treated with GnRH agonists, though these findings were only applicable to hormone receptor-negative women (Li *et al.* 2022). Thus, despite the emergence of some prospective data, evidence of the benefit of GnRH agonists for fertility preservation remains uncertain and limited.

## Current avenues in oncofertility research

Considerable research has been conducted in recent years to discover new, more effective fertility preservation strategies which can prevent ovarian damage and preserve oocytes *in vivo*. Technologies such as whole ovarian transplantation, oocyte *in vitro* growth and maturation and development of artificial ovaries are currently under investigation (Kim *et al.* 2021, McClam & Xiao 2022). Additionally, stem cell treatment is another emerging area of research, with several studies investigating the possibility that administration of mesenchymal stem cells or stem cell-derived factors might improve ovarian function post-cyclophosphamide or cisplatin treatment in murine models (Hong *et al.* 2020, Çil & Mete 2021, Salvatore *et al.* 2021, Shin *et al.* 2021, Bahrehbar *et al.* 2022, Liu *et al.* 2022). However, the remainder of this review will focus on current avenues of research aimed at preventing follicle loss after damage induced by alkylating and alkylating-like agents, utilising adjuvant ferto-protective agents aimed at (i) preventing apoptosis of ovarian follicles or (ii) blocking overactivation of primordial follicles. Though, when interpreting the results of the following studies, it is important to consider the dose of the ferto-protective agent utilised as well as the ratio of this to the chemotherapy drug.

## Preventing apoptosis of primordial follicles

Given that direct DNA damage and subsequent apoptosis appears to be a major mechanism behind cyclophosphamide- and cisplatin-induced primordial follicle loss, considerable research has been conducted into agents that may prevent oocyte apoptosis from occurring. These can be separated into two major categories: (i) protein kinase inhibitors and (ii) ceramide-induced death pathway inhibitors (Table 3). Additionally, the administration of luteinising hormone has shown some efficacy in preventing primordial follicle loss and preserving long-term fertility *in vivo* in mice following exposure to cyclophosphamide and cisplatin (Rossi *et al.* 2017, Del Castillo *et al.* 2021).

**Table 3:** Summary of current avenues of research into ferto-protective agents to prevent chemotherapy-induced ovarian damage.

Aim	Class/agent	Mechanism	Target agent	Evidence of primordial follicle protection
Block apoptosis of primordial follicles				
Protein kinase inhibitors				
Imatinib		Prevents TAp63α activation and subsequent apoptosis via inhibition of c-ABL	Cisplatin	Conflicting evidence regarding efficacy and rationale behind mechanism of protection (Gonfloni *et al.* 2009, Kerr *et al.* 2012*a*, Kim *et al.* 2013, Morgan *et al.* 2013); Shown to actually induce primordial follicle apoptosis in mice and humans (Bildik *et al.* 2018, Salem *et al.* 2020)
Asciminib		Prevents TAp63α activation and subsequent apoptosis *via* inhibition of c-ABL	Cyclophosphamide	Evidence of efficacy *in vivo* in mice; however, follicle quantification method utilised may be insufficient (Mattiello *et al.* 2021)
CK2II, ETP46464		Prevents TAp63α activation and subsequent apoptosis *via* inhibition of CHK2 and ATR	Cyclophosphamide	Evidence of efficacy *in vitro* in mice (Luan *et al.* 2019) but no *in vivo* evidence of efficacy
KU55933		Prevents TAp63α activation and subsequent apoptosis *via* inhibition of ATM	Cyclophosphamide	Evidence of efficacy *in vitro* in mice (Ganesan & Keating 2016) but no *in vivo* evidence of efficacy
Inhibitors of ceramide-induced apoptosis		Prevents the activation of apoptosis via inhibition of ceramide	Cyclophosphamide	
S1P				Effective in murine human ovarian xenograft models (Meng *et al.* 2014, Li *et al.* 2017) Ineffective in rats *in vivo* (Kaya *et al.* 2008)
C1P				Evidence of efficacy *in vivo* in mice (Pascuali *et al.* 2018)
Endogenous hormones				
LH		Stimulates release of anti-apoptotic signals from somatic cells possessing LH receptors	Cyclophosphamide, cisplatin	Evidence of efficacy *in vivo* in mice following cisplatin treatment (Rossi *et al.* 2017); mild protection against primordial follicle loss and preservation of long-term fertility *in vivo* in mice following cyclophosphamide, though small sample sizes were utilised (Del Castillo *et al.* 2021)
Prevent overactivation of primordial follicles				
PI3K pathway inhibitors		Prevent primordial follicle activation *via* inhibition of PI3K pathway activity		
AS101			Cyclophosphamide	Evidence of efficacy *in vivo* in mice, although only a short-term breeding study was performed (Kalich-Philosoph *et al.* 2013).
Melatonin			Cisplatin	Evidence of efficacy *in vivo* in mice; however, follicle quantification methods utilised may be insufficient and long-term fertility was not assessed (Jang *et al.* 2016, 2017)
Rutin			Cisplatin	Evidence of efficacy *in vivo* in mice, although fertility was not assessed (Lins *et al.* 2020)
mTOR pathway inhibitors		Prevent primordial follicle activation *via* inhibition of mTOR pathway activity		
RAD001, INK128			Cyclophosphamide, cisplatin	Evidence of efficacy *in vivo* in mice; however, follicle quantification method utilised may be insufficient and long-term fertility was not assessed (Goldman *et al.* 2017, Tanaka *et al.* 2018)
Rapamycin			Cyclophosphamide	Evidence of efficacy *in vivo* in mice, although long-term fertility was not assessed (Zhou *et al.* 2017)
Endogenous hormones				
rAMH		Prevents primordial follicle activation *via* administration of AMH, a regulator of primordial follicle activation	Cyclophosphamide	Mixed results regarding the protection of primordial follicles in several *in vivo* murine studies (Kano *et al.* 2017, Roness *et al.* 2019, Sonigo *et al.* 2019); inconclusive efficacy in preserving long-term fertility in mice (Roness *et al.* 2019, Sonigo *et al.* 2019)
Reduce oxidative stress				
Antioxidants		Prevent oxidative stress-induced stromal and vascular damage by eliminating reactive oxygen species, thus preventing primordial follicle loss indirectly		
Bilberry			Cisplatin	Some evidence of ovarian protection reported in one *in vivo* murine study, although primordial follicles were not quantified and fertility was not assessed (Pandir *et al.* 2014)
Sildenafil citrate			Cisplatin	Evidence of efficacy *in vivo* in mice; however, follicle quantification methods utilised may be insufficient and fertility was not assessed (Taskin *et al.* 2015)
Hydrogen-rich saline			Cisplatin	Some evidence of ovarian protection reported in one *in vivo* murine study, although primordial follicles were not quantified and fertility was not assessed (Meng *et al.* 2015)
Mirtazapine, hesperidin			Cyclophosphamide	Higher rate of fertility reported in 6-month breeding study in mice, although no other fertility parameters were analysed (Altuner *et al.* 2013); evidence of efficacy *in vivo* in mice; however, follicle quantification methods utilised may be insufficient (Khedr 2015)
Puerarin			Cyclophosphamide	Evidence of efficacy *in vivo* in mice, although fertility was not assessed (Chen *et al.* 2021)
Resveratrol			Cyclophosphamide	Evidence of efficacy *in vivo* in mice, although fertility was not assessed (Chhabria *et al.* 2022)

AS101, ammonium trichloro(dioxyethylene-o,o’)tellurate; ATM, ataxia–telangiectasia mutated; ATR, ataxia–telangiectasia and Rad3 related; CHK2, checkpoint kinase 2; LH, luteinising hormone; mTOR, mammalian target of rapamycin; PI3K, phosphoinositide 3-kinase; rAMH, recombinant anti-Müllerian hormone; SIP, sphingosine-1-phosphate.

### Protein kinase inhibitors

#### Imatinib

Imatinib is a selective inhibitor of breakpoint cluster region (BCR)-Abelson murine leukemia viral oncogene homolog 1 (ABL), a tyrosine kinase produced by the abnormal fusion of two genes – *BCR* and cellular-ABL (*c-ABL*). This chimeric gene, termed Philadelphia chromosome, is strongly associated with the development of leukaemia. Inhibition of BCR-ABL prevents apoptosis by blocking mitochondrial cytochrome c release and caspase activation (Deming *et al.* 2004). Additionally, imatinib is also able to inhibit c-ABL, which is associated with induction of the intrinsic apoptotic pathway *via* the activation of p73 (Yuan *et al.* 1999). This could, theoretically, downregulate TAp63-mediated apoptosis within primordial follicles.

Initially, imatinib was reported to prevent cisplatin-induced primordial follicle apoptosis in mice both *in vitro* and* in vivo* (Gonfloni *et al.* 2009). Similar results were obtained in two subsequent *in vitro* studies (Kim *et al.* 2013, Morgan *et al.* 2013). However, other studies found opposing results, showing that imatinib conferred no protection from cisplatin-induced apoptosis *in vivo* in wild-type mice (Kerr *et al.* 2012*a*) or in mice bearing human ovarian cortical xenografts (Bildik *et al.* 2018) nor *in vitro* in human ovarian cortical samples (Bildik *et al.* 2018). However, in the latter case, it is important to consider that the imatinib dose used *in vitro* may equate to a higher dose than what was utilised in the aforementioned *in vivo* studies. Additionally, subsequent work in reply to Kerr *et al.* corroborated the initial findings by Gonfloni *et al.* (Maiani *et al.* 2012). The discrepancy in these two reports may be due to differences in the brands of cisplatin utilised and their respective efficacies in inducing cell death (Maiani *et al.* 2012). After repeating some key experiments using an equivalent cisplatin dose from Gonfloni *et al.* but utilising the same cisplatin brand as Kerr *et al.,* Maiani *et al.* found that imatinib treatment protected oocytes from primordial and primary follicles from cisplatin both *ex vivo* and *in vivo.* It is possible that imatinib can protect oocytes at lower doses of cisplatin (Gonfloni *et al.* 2009, Maiani *et al.* 2012) but is unable to rescue damage at higher doses (Kerr *et al.* 2012*a*). This highlights that the dose of chemotherapy, as well as the ratio of chemotherapy to the ferto-protective agent utilised, must be taken into consideration when designing and interpreting studies aimed at preventing chemotherapy-induced ovarian damage.

On the contrary, it was more recently found that c-ABL was not activated in response to cisplatin treatment, which may explain why imatinib may not in fact prevent primordial follicle apoptosis. Indeed, imatinib treatment may actually result in primordial follicle depletion and increased atresia (Bildik *et al.* 2018). This is the first molecular evidence that imatinib may in fact be ovotoxic to humans albeit in culture. A recent murine study corroborated this idea, showing that *in vivo* imatinib administration depletes primordial follicles and reduces embryo quality in mice (Salem *et al.* 2020). However, earlier studies have indicated that imatinib does not induce ovarian toxicity (Maiani *et al.* 2012). Thus, it remains unclear and contentious if imatinib is an effective fertility preservation strategy and may in fact be detrimental to ovarian function.

#### Asciminib

Asciminib is an allosteric inhibitor of c-ABL. A recent study showed that administration of asciminib can protect ovarian follicles from cyclophosphamide-induced apoptosis (Mattiello *et al.* 2021). However, this study utilised a suboptimal follicle quantification method in which the total number of follicles within the ovary was not counted and only mid-ovary sections were analysed. Furthermore, the number of primordial and primary follicles was reported together and not separately, making it difficult to determine the precise effects of asciminib on the ovarian reserve. Thus, further work is required to determine the utility of asciminib as an ovary protectant.

#### CK2II and ETP46464

Ataxia–telangiectasia and Rad3-related (ATR) and checkpoint kinase 2 (CHK2) are both serine–threonine kinases that have important roles in the DNA damage response. Following the induction of DNA damage, ATR activates CHK2 which, in turn, can activate TAp63 and trigger apoptosis within oocytes (Rinaldi *et al.* 2019). It has been shown that CHK2-deficient mice are resistant to irradiation-induced oocyte loss in mice, and *in vitro* inhibition of either CHK2 or CK1 is able to block oocytes from *TAp63-*mediated apoptosis caused by radiation, cisplatin, cyclophosphamide or doxorubicin (Kim *et al.* 2013, Tuppi *et al.* 2018, Luan *et al.* 2019, Rinaldi *et al.* 2019). CK2II (a CHK2 inhibitor) and ETP46464 (an ATR inhibitor) were both able to prevent primordial follicle apoptosis in cultured mouse ovaries *in vitro* from 4-hydroxyperoxycyclophophamide, a cyclophosphamide metabolite (Luan *et al.* 2019). Levels of phosphorylated TAp63α within exposed primordial follicle oocytes were barely detectable following CK2II and ETP46464 administration. Although promising, the *in vivo* efficacy of these inhibitors for fertility preservation is yet to be determined, and side effects need to be thoroughly assessed.

#### KU55933

ATM is a key mediator of the DNA damage response. In oocytes, when DNA damage is irreparable, ATM can induce apoptosis by activating TAp63 either directly or indirectly *via* CHK1/2 (Ou *et al.* 2005, Tuppi *et al.* 2018). Ganesan *et al.* found that KU55933, an ATM inhibitor, prevents primordial follicle loss *in vitro*, in cultured rat ovaries following exposure to phosphoramide mustard, a cyclophosphamide metabolite (Ganesan & Keating 2016). Currently, there is no *in vivo* evidence of the effectiveness of KU55933 in preventing primordial follicle apoptosis, and the important role for ATM in coordinating the DNA damage response in other cells, may preclude its use as a fertility preservation agent unless targeted delivery could be achieved.

### Inhibitors of ceramide-induced apoptosis

Ceramides play key roles in both the intrinsic and extrinsic apoptotic pathways, and ceramide-induced apoptosis has been linked to the age-related decline in primordial follicles (Perez *et al.* 2005). Sphingosine-1-phosphate (S1P) and ceramide-1-phosphate (C1P) are endogenous sphingolipids which are natural inhibitors of ceramide-induced apoptosis.

#### Sphingosine-1-phosphate

S1P has previously been shown to prevent cyclophosphamide-induced primordial follicle loss in mice, *in vitro* and *in vivo* (Morita *et al.* 2000, Hancke *et al.* 2007). In a murine human ovarian xenograft model, administration of S1P was shown to prevent apoptosis in human primordial follicles in response to cyclophosphamide, as evidenced by a significantly reduced number of primordial follicles positive for activated caspase-3 (Li *et al.* 2014). However, an earlier study found no difference in the level of active caspase-3 or TUNEL-positive primordial follicles between cyclophosphamide-exposed S1P-treated rats compared with controls (Kaya *et al.* 2008). Importantly, both of these studies did not quantify the overall number of primordial follicles within the ovaries, making it difficult to fully assess the efficacy of S1P in protecting the entire primordial follicle pool. A later mouse xenograft study utilising human fetal ovaries reported that S1P treatment did significantly protect primordial follicles from cyclophosphamide (Meng *et al.* 2014).

#### Ceramide-1-phosphate

A recent mouse study demonstrated that C1P delivered intrabursally protects the ovarian reserve and prevents primordial follicle apoptosis following cyclophosphamide exposure (Pascuali *et al.* 2018). Additionally, *in vitro* maturation and IVF studies showed that C1P administration preserves oocyte quality. A short-term, two-round breeding study was also conducted, which showed fertility was also preserved. Lastly, gross uterine morphology and endometrial structure appeared normal, suggesting C1P may also protect from uterine damage. Although promising, evidence of the efficacy of C1P for fertility preservation is limited to this study only.

## Blocking overactivation of primordial follicles

Although still not fully understood, primordial follicle activation is thought to be mediated by induction of PI3K and mTOR signalling – the latter of which acts downstream of PI3K – as well as loss of inhibitory signals, such as AMH (Chen *et al.* 2020). In addition to GnRH agonists, several other agents have been investigated in recent years with the aim of blocking overactivation of primordial follicles. These can be divided into three categories: (i) PI3K pathway inhibitors; (ii) mTOR pathway inhibitors; and (iii) glycoprotein hormones.

### PI3K and mTOR pathway inhibitors

#### AS101

Ammonium trichloro(dioxyethylene-*o,o*’)tellurate (AS101) is an immunomodulator that has also been shown to inhibit PI3K pathway activity (Hayun *et al.* 2006). In the initial publication which proposed the ‘burnout’ theory, Kalich-Philosoph *et al.* also reported that AS101 is able to suppress primordial follicle activation and, thus, protect the ovarian reserve following cyclophosphamide exposure (Kalich-Philosoph *et al.* 2013). Co-treatment with AS101 resulted in a significant increase in primordial follicle survival when compared to mice exposed only to cyclophosphamide. A breeding study revealed that AS101 co-treated mice also appeared more fertile, producing significantly more pups per litter and cumulative pups compared to cyclophosphamide-treated mice. However, mice only underwent three breeding rounds, and thus it remains unclear whether AS101 administration can effectively preserve the fertile life span following cyclophosphamide treatment.

#### Melatonin

Melatonin is an endogenous hormone produced by the pineal gland, which has antioxidant properties and is able to suppress PI3K pathway activity. Two studies, from the same group, report that melatonin prevents cisplatin-induced primordial follicle depletion (Jang *et al.* 2016, 2017). However, follicle quantification was conducted on only three 5 µm ovarian sections per animal, which is likely insufficient to accurately estimate the number of primordial follicles within the whole ovary. Long-term fertility was also not assessed in either study. Similarly, a recent study indicated that melatonin prevents cyclophosphamide-induced POI in rats, although only total follicle numbers were reported and fertility was not assessed (Xu *et al.* 2022).

#### RAD001 and INK128

RAD001 is an mTOR complex (mTORC) 1 inhibitor, and INK128 inhibits mTORC1/2. A recent report showed that the administration of RAD001 or INK128 prevents the depletion of primordial follicles following cyclophosphamide treatment (Goldman *et al.* 2017). One of the limitations of this study is the use of density to quantify follicles, as this method does not account for the uneven distribution of primordial follicles within the whole ovary nor for differences in ovarian volume between groups. Follicle density may therefore not reflect actual numbers (Winship *et al.* 2020). Encouragingly, RAD001- and INK128-treated mice produced significantly more pups per litter after a single round of mating. In addition, another recent study reported that RAD001 administration protects from cisplatin-induced primordial follicle depletion (Tanaka *et al.* 2018), although a breeding study was not conducted. Thus, whether RAD001 or INK128 preserves the length of the fertile life span remains to be seen following cyclophosphamide or cisplatin exposure.

#### Rapamycin

Rapamycin is an allosteric inhibitor of mTOR, which has been shown to prevent cyclophosphamide-induced primordial follicle depletion in mice (Zhou *et al.* 2017). A breeding study was not conducted; thus, it is yet to be determined whether the administration of rapamycin can effectively preserve long-term fertility following cyclophosphamide. Additionally, PI3K/mTOR pathways have other complex roles within cells independent of primordial follicle activation, such as cell death and survival (Sarbassov *et al.* 2005, Steelman *et al.* 2011), that require further consideration when interpreting these data.

### Endogenous hormones

#### Recombinant AMH

There is evidence to suggest that AMH is involved in suppressing primordial follicle activation and, thus, maintaining their quiescence, which has led to the hypothesis that administration of human recombinant AMH (rAMH) can prevent chemotherapy-mediated follicle loss. This concept was first investigated by Kano *et al.* who reported a significant increase in primordial follicles following cyclophosphamide exposure in mice treated with rAMH, when compared to controls (Kano *et al.* 2017). Although not discussed, follicle depletion was still extensive in cyclophosphamide exposed rAMH-treated mice compared to controls; thus, the level of protection is unclear, and long-term fertility was not assessed.

Another study reported that rAMH administration prevented cyclophosphamide-induced primordial follicle depletion; however, robust results were only obtained when primordial follicle number was expressed as a proportion of total follicles within the ovary and not in the raw follicle counts (Sonigo *et al.* 2019). A short breeding study was conducted, but it is not clear if fertility was improved. Interestingly, however, rAMH administration significantly increased the number of mature oocytes retrieved after superovulation, which is an exciting avenue to follow-up in future studies. A similar study by the same group reported a significant increase in primordial follicles in cyclophosphamide-exposed mice treated with rAMH (Roness *et al.* 2019). Fertility studies showed more promising results, with a significantly increased pregnancy rate in rAMH-treated mice over five breeding rounds. Although the breeding study ceased prior to the loss of fertility of the mice, and thus the entire fertile life span was not captured, these studies collectively suggest that rAMH may be an avenue worth investigating further.

## Future perspectives

### Chemotherapy may also alter uterine function

The unintended side effects of chemotherapy treatment on the ovary have been a major focus of research thus far. It is clear that chemotherapy treatment – particularly alkylating agents – can significantly impact a woman’s ovarian reserve and, by extension, fertile life span. However, the possibility of chemotherapy-induced damage to the remainder of the reproductive tract, including the uterus, has received considerably less attention.

The integrity of the uterus and, in particular, the endometrium is fundamental to the establishment and maintenance of successful pregnancy. The impact of and mechanisms behind radiotherapy-induced uterine damage on pregnancy success in female cancer survivors are incompletely characterised and have been recently reviewed (Garg *et al.* 2020, Griffiths *et al.* 2020). Additionally, there is emerging evidence to suggest that the uterus may be an additional site of chemotherapy-induced damage in women.

However, the nature and impact of chemotherapy-induced uterine injury are poorly understood. Information in the literature is sparse and restricted mostly to retrospective human studies, which are confounded by many inherent limitations and inconsistencies. These are mostly due to the lack of information available regarding treatment regimens used, including which agent(s) were used and whether combination therapies were administered. Moreover, ascertaining whether pregnancy outcomes in women have been impacted by chemotherapy-induced uterine-specific damage is often confounded by the ovarian and endocrine damage that occurs concurrently. Future studies in this area are critical to furthering our understanding of the impact of chemotherapy on the uterus and what mechanisms may underlie it. Furthermore, addressing this knowledge gap will be essential in the development of fertility preservation strategies specific to the uterus.

### Novel, non-cytotoxic agents

Although chemotherapies such as cyclophosphamide and cisplatin remain a mainstay of many cancer treatment regimes, the use of more targeted therapies – such as immunotherapy and small-molecule inhibitors – is becoming more widespread. However, information on whether these newer therapies may also impact ovarian function and fertility, even in preclinical models, is somewhat limited. Recently, we and others critically reviewed the available literature and speculated on the potential impacts of immunotherapies and small-molecule inhibitors on reproductive and endocrine function in women (Alesi *et al.* 2021, Garutti *et al.* 2021, Lambertini *et al.* 2022, Rosario *et al.* 2022). We also recently demonstrated that a prominent class of immunotherapy – immune checkpoint inhibitors – causes profound ovarian dysfunction in a mouse model (Winship *et al.* 2022). This damage is likely to be permanent and may impact fertility, as a significant reduction of primordial follicles was found – a concerning issue as these drugs are already in widespread clinical use and reproductive endpoints are rarely assessed during clinical trials (Cui *et al.* 2021). A recent study looking at fertility-related prescribing information available for 32 novel non-cytotoxic cancer agents approved for use in the USA and Australia found that only four listed recommendations regarding potential human fertility risks (Volckmar *et al.* 2022). Clearly, investigation into the ovotoxicity of newer treatments, such as immunotherapy and other targeted therapies, must be prioritised.

### Extending oncofertility research beyond animal models

Research conducted in animal models has greatly underpinned the field of oncofertility to date, given that measuring the ovotoxic effects of chemotherapy in women is difficult. This is because clinical data from women is often complicated by the fact that chemotherapies are generally administered in tailored multi-agent, multi-dose regimens, making it difficult to compare findings between different patients and to define the precise impacts of individual agents on ovarian function. Additionally, analysis of the whole ovary is generally not possible nor practical; and there are currently no methods available to directly assess the ovarian reserve of primordial follicles. Therefore, in a clinical setting, ovarian function must be investigated through alternative, indirect measures, such as assessing patient menstrual history, measuring ovarian volume or performing antral follicle counts or using surrogate endocrine markers, such as circulating AMH levels. Thus, investigation of the effects of chemotherapies and ferto-protective agents on the whole ovary utilising animal models, such as the mouse, remains imperative (Winship *et al.* 2020). It is also important to consider that the aforementioned clinical methods do not provide any insight into oocyte quality (Winship *et al.* 2020), further reinforcing the need for rigorous studies in animal models in order to fully characterise the long-term implications of chemotherapy treatment and ferto-protective agents on fertility and offspring health.

Despite these benefits and the fact that ovarian morphology, function and aging share many similarities in humans and rodents (Coxworth & Hawkes 2010, Cunha *et al.* 2019), rodent models do have some important limitations to consider. This includes their differences in life span, number of ovulations per cycle and the fact that they do not menstruate or experience menopause. Therefore, it is important that the field moves towards performing more studies in non-human primates and utilising human tissue more frequently, such as culturing human cortical pieces *in vitro* and establishing *in vivo* human ovarian xenograft animal models. However, access to human tissue is a significant barrier that must be improved in order to make this a reality. Additionally, the development of human ovary organoids is an exciting prospect that could also be highly beneficial. Furthermore, it is critical that further research be conducted to discover a direct biomarker of primordial follicles, to allow for more accurate measurement of the ovarian reserve in women.

## Conclusions

It has been known for many years that traditional cytotoxic cancer treatments, like cyclophosphamide and cisplatin, can damage the ovary and compromise fertility. Much research now focuses on defining the cellular and molecular mechanisms that underlie this ovarian damage, as this knowledge is critical for the development of effective fertility preservation agents aimed at preventing follicle depletion to preserve endogenous endocrine function and fertility. In addition, understanding the extent to which new precision cancer treatments, such as immunotherapies, compromise ovarian function is now emerging as a priority for researchers, clinicians and patients alike. Whilst the ovary has been the focus of studies concerning cancer treatment and infertility, moving forward, assessing impacts of existing and emerging cancer treatments on the uterus may be important for maximising pregnancy outcomes in female cancer survivors.

## Declaration of interest

The authors declare that there is no conflict of interest that could be perceived as prejudicing the impartiality of the research reported.

## Funding

This work was made possible through Victorian State Government Operational Infrastructure Support and Australian Government NHMRC IRIISS. Additionally, this work was supported by funding from the Australian Research Council (ARC); KJH – FT190100265 and ALW – DE21010037, the National Health and Medical Research council (NHMRC); JMS-2011299, and the Australian National Breast Cancer Foundation (NBCF) grant no. IIRS-22-092. LRA is supported by an Australian Government Research Training Program scholarship and a Monash Graduate Excellence Scholarship. Figures were created using BioRender.

## Author contribution statement

LRA designed tables and figures. All authors contributed to writing and editing the manuscript.
